# Circular RNA hsa_circ_0007121 regulates proliferation, migration, invasion, and epithelial–mesenchymal transition of trophoblast cells by miR-182-5p/PGF axis in preeclampsia

**DOI:** 10.1515/med-2020-0230

**Published:** 2020-10-14

**Authors:** Shukun Gai, Li Sun, Huiying Wang, Ping Yang

**Affiliations:** Department of Obstetrics, Yantai Yuhuangding Hospital, 20 Yudong Road, Zhifu District, Shandong Province, 264000, Yantai, Shandong, China

**Keywords:** PE, hsa_circ_0007121, miR-182-5p, PGF

## Abstract

**Background:**

Mounting evidence has revealed that abnormal expression of circular RNAs play pivotal roles in many human diseases including preeclampsia (PE). While human sapiens circular RNA 0007121 (hsa_circ_0007121) has been verified to be downregulated in human placental tissues, the underlying mechanisms were still unclear. This research aims to investigate the effect and underlying mechanisms of hsa_circ_0007121 in preeclampsia.

**Methods:**

The expression of hsa_circ_0007121, microRNA (miR)-182-5p, and placental growth factor (PGF) was assessed by quantitative reverse transcription polymerase chain reaction in PE placentas relative to the expression in normal pregnancy placentas. After transfection, cell counting kit-8 assay was employed to detect cell proliferation. Cell migration and invasion were tested by the transwell assay. The relative level of epithelial–mesenchymal transition (EMT)-related proteins in HTR-8/SVneo cells and PGF in placentas samples were measured by western blot. The relationship between miR-182-5p and hsa_circ_0007121 or PGF was predicated by circular RNA interactome or ENCORI and verified by dual-luciferase reporter assay and RNA immunoprecipitation assay.

**Results:**

The levels of hsa_circ_0007121 and PGF were significantly declined in PE placental tissues and HTR-8/SVneo cells, whereas miR-182-5p had an opposite result. Downregulation of hsa_circ_0007121 obviously inhibited HTR-8/SVneo cell proliferation, migration, invasion, and EMT, while upregulation of hsa_circ_0007121 promoted this process. Besides, miR-182-5p was a target gene of hsa_circ_0007121 and could target PGF. Further analysis indicated that hsa_circ_0007121 regulated the proliferation, migration, invasion, and EMT of HTR-8/SVneo cells via altering PGF expression by interacting with miR-182-5p.

**Conclusion:**

Hsa_circ_0007121 mediated the progression of PE via miR-182-5p/PGF axis.

## 1 Introduction

Preeclampsia (PE) affected 2–8% of pregnancies worldwide and led to 46,900 deaths in 2015 [1]. Hypertension, diabetes mellitus, proteinuria, obesity, family history, multiple pregnancies, and thrombotic vascular disease are the risk factors for PE [2]. Previous studies showed that the inadequate trophoblast invasion was correlated with PE [3,4,5]. Also, growing evidence indicated that epithelial–mesenchymal transition (EMT) was related to the development of PE [6,7]. HTR-8/SVneo cell line is human being chorial trophocyte cell that was always used for the study of trophoblast biology and placental function, which may improve our understanding of diseases related to tumor progression, abnormal placentation hypoinvasiveness in preeclampsia, and hyperinvasiveness in trophoblastic neoplasms [8,9]. Although the potential pathogenesis of PE is barely elucidated, we chose HTR-8/Svneo cell as a study subject *in vitro*.

Circular RNAs (CircRNAs) could accumulate in specific cell types in a temporally regulated manner owing its high stability, which was presumably the result of their covalently closed ring structure protecting these molecules from exonuclease-mediated degradation [10]. Increasing evidence has suggested that CircRNAs play a vital role in many diseases including PE. Garikipati et al. reports indicate that CircFndc3b modulated cardiac repair after myocardial infarction via FUS/VEGF-A axis [11]. Holdt et al. found that circRNA antisense noncoding RNA in the INK4 locus (ANRIL) modulated ribosomal RNA maturation and atherosclerosis [12]. Furthermore, recent reports indicated that circRNAs functioned in regulating PE progression [13,14,15]. Researchers attempted to investigate the profile of circRNAs in placental tissues of preeclampsic women and also examined the potential effects of circRNAs dysregulation on the progression of PE. From a total of 22,796 circRNAs, Bai et al. identified 300 differentially expressed circRNAs and found that the potential noninvasive biomarker hsa_circ_0007121, which could help to predict PE [16]. Thus, hsa_circ_0007121 is a noninvasive biomarker for the prediction of PE, which still needs further investigation due to its uncharted mechanisms.

MicroRNAs (miRNAs) are a type of small RNAs (about 22 nucleotides), and they combine with messenger RNAs (mRNAs) in the 3′-untranslated region (3′UTR) to modulate its expression [17]. Emerging reports manifested that miRNAs played a pivotal role in a variety of pregnancy-related complications such as preeclampsia and fetal growth restriction [18]. Lv et al. uncover that miR-145-5p facilitated the trophoblast cell growth and invasion via targeting FLT1 [19]. Yuan et al. found that miR-16 regulated the pathogenesis of PE via targeting Notch2 [20]. More recently, Fang et al. confirmed that the upregulated miR-182-5p promotes PE progression [21]. MiR-182, a precursor to miR-182-5p, also linked to altered angiogenesis in PE [22]. Yet, it is very significant to clarify the role of different miRNAs in orchestrating the placental vascular development.

The placental growth factor (PGF) belongs to the vascular endothelial growth factor (VEGF) family. The overexpression of VEGF is linked to trophoblastic failed invasion, which was widely accepted as one of the PE key factors [23,24]. PGF can regulate angiogenesis, which is important for the development of the embryo [25]. PGF levels were found significantly lower during PE, and its levels correlated with the severity of the disease, which was possible to predict the development of PE [26,27,28,29,30]. Wu et al. found that decreased PGF might lead to trophoblast dysfunction in fetal growth restriction [31]. Kurtoglu et al. reported that serum PGF might be a significant marker to predict the severity of PE [32]. Besides, Gao et al. reported that PGF was clearly downregulated in PE placental tissues [33]. Therefore, PGF could be a potential target, and corresponding regulators should be explored.

In our study, we checked the levels of hsa_circ_0007121, miR-182-5p, and PGF in PE placental tissues and HTR-8/SVneo cells. In addition, the role and the possible regulatory mechanism of hsa_circ_0007121 in PE were also studied.

## 2 Material and methods

### 2.1 Samples and cell culture

Thirty-five patients with PE and 35 gestational and maternal age-match healthy women were included in this study. PE diagnosis was according to American College of Obstetricians and Gynecologists 2013 diagnostic criteria [34], with systolic blood pressure more than 140 mm Hg or diastolic blood pressure more than 90 mm Hg, either accompanied by proteinuria or edema. The subjects were limited to nulliparous women with a singleton pregnancy at 20 + 0 to 24 + 6 weeks gestation. Exclusion criteria were as follows: underlying medical disease, previous cervical surgery, history of pregnancy losses, known fetal abnormality or abnormal karyotype, or accepted obstetric intervention at recruitment. The PE placental tissues (*n* = 35) and normal placental tissues (*n* = 35) were collected from participants at Yantai Yuhuangding Hospital (Yantai, China) between March 2017 and October 2019. Each participant signed the informed consent, and this research was authorized by the Ethics Committee of Yantai Yuhuangding Hospital. Placental samples were taken from a representative block of the central portion of tissue below one-third of the placenta near maternal side and preserved in a freezer at −80°C for later use. The human trophoblast cells (HTR-8/SVneo) were purchased from American Type Culture Collection (Manassas, VA, USA) and then was cultivated in the McCoy’s 5A medium (Sigma, St Louis, MO, USA) with 10% fetal bovine serum (FBS; Sigma) and 5% CO_2_. Transcription inhibition experiment was performed by adding 2 μg/mL actinomycin D (Sigma) to the medium, and dimethylsulphoxide (DMSO; Sigma) was used as the control.

### 2.2 Cell transfection

Small interfering RNA for hsa_circ_0007121 (si-hsa_circ_0007121), miR-182-5p mimic (miR-182-5p), miR-182-5p inhibitor (anti-miR-182-5p), small interfering RNA for PGF (si-PGF), and the controls (si-NC, NC, anti-NC, and scramble) were sourced from GenePharma (Shanghai, China). Hsa_circ_0007121 overexpression plasmid (named as hsa_circ_0007121), PGF overexpression plasmid (PGF), and corresponding matched controls (circ-NC and vector) were acquired from RiboBio (Guangzhou, China). Lipofectamine 3000 (Solarbio, Beijing, China) was purchased from Sigma and used to transfect cells following the provided procedures.

### 2.3 Quantitative reverse transcription polymerase chain response (qRT-PCR) and RNase treatment

The TRIzol reagent (Sigma) was applied for RNA extraction, and PrimeScript RT Master Mix kit (Takara, Dalian, China) was used for reverse transcription. Then, the QuantiTect SYBR Green RT-PCR Kit (Qiagen, Shanghai, China) was used to perform the qRT-PCR for hsa_circ_0007121 and PGF. The miScript SYBR Green PCR kit (Qiagen) was used for the qRT-PCR of miR-182-5p. Beta-actin (β-actin) was used to normalize hsa_circ_0007121 and PGF expression, and U6 was used to normalize miR-182-5p expression. The data were computed using the 2^−ΔΔCt^ method. The following primers were used (5′ to 3′): hsa_circ_0007121 (F, GGGGGTTTTATTTCAGGTGGA; R, AGGGGAAAAATAGTCCTCACAGA); linear mRNA primer (F, AGTTTTAGGCGTGGCTGTGA; R, CACGATTGCTCACAATGGAGG); miR-182-5p (F, ATCACTTTTGGCAATGGTAGAACT; R, TATGGTTTTGACGACTGTGTGAT); PGF (F, CCCACCTGGATGCTGTT; R, ATAGAGGGTAGGTACCAG); β-actin (F, GCACCACACCTTCTACAATG; R, TGCTTGCTGATCCACATCTG); U6 (F, TCCGGGTGATGCTTTTCCTAG; R, CGCTTCACGAATTTGCGTGTCAT). RNase R (Sigma) was utilized to treat purified RNAs to check the circular form of hsa_circ_0007121.

### 2.4 Cell counting kit-8 (CCK-8) assay

HTR-8/SVneo cells were seeded into a 96-well plate and added with 10 µL CCK-8 solution (MedChemExpress, Shanghai, China). After 2 h, the optical density at 450 nm wavelength was checked with a microplate reader (Bio-Rad, Richmond, Virginia, USA).

### 2.5 Transwell assay

Transwell chamber precoated with or without Matrigel (Solarbio) was utilized to evaluate cell invasion or migration, respectively. Cells with serum-free medium were added into the upper chamber, and medium containing fetal bovine serum was added into the lower chamber. Then, the cells were treated with crystal violet (Solarbio) and were analyzed using the microscope (MTX Lab Systems, Bradenton, FL, USA).

### 2.6 Western blot

Western blot was executed according to the previous report [12]. Briefly, after the extraction and separation, proteins were incubated with the primary antibodies and the secondary antibody. The protein band was observed using ECL kit (Solarbio). Antibodies used in this research were as follows: anti-E-cadherin (1:1,000, ab15148, Abcam, Cambridge, United Kingdom), anti-Vimentin (1:3,000, ab137321, Abcam), anti-snail (1:1,000, ab82846, Abcam), anti-N-cadherin (1:2,500, ab18203, Abcam), anti-matrix metalloprotein (MMP)-2 (1:3,000, ab97779, Abcam), anti-MMP-9 (1:1,000, ab38898, Abcam), anti-PGF (1:2,500, ab196666, Abcam), anti-glyceraldehyde 3-phosphate dehydrogenase (1:2,500, ab9485, Abcam), and Goat Anti-Rabbit IgG H&L (HRP) (1:3,000, ab205718, Abcam).

### 2.7 Dual-luciferase reporter assay

The potential target sequences in hsa_circ_0007121 or PGF of miR-182-5p were predicated by CircRNA interactome or ENCORI, respectively. The sequence of hsa_circ_0007121 or PGF 3′UTR was inserted into pGL3 vector (Promega, Madison, WI, USA) for the establishment of hsa_circ_0007121-wt or PGF-wt reporter vector. Also, the hsa_circ_0007121-mut or PGF-mut reporter vector was constructed by mutating the possible binding sites. Then, HTR-8/SVneo cells were cotransfected with reporter vector and miR-182-5p or miR-NC. The luciferase activity was checked by using the Dual-Glo^®^ Luciferase Assay System kit (Promega).

### 2.8 RNA immunoprecipitation (RIP) assay

Magna RIP RNA-Binding Protein Immunoprecipitation Kit (Millipore, Billerica, MA, USA) was introduced for RIP in line with the given protocols. In brief, cells were lysed and incubated with anti-Argonaute 2 antibody (Anti-Ago2; Millipore) with conjugated magnetic beads for 24 h, and then, the beads were treated with proteinase K to remove protein. The immunoglobulin G (IgG) was used as a control. The immune precipitated RNA was purified and analyzed by qRT-PCR.

### 2.9 Statistical analysis

Experimental data were presented by mean ± standard deviation and analyzed by GraphPad Prism (GraphPad, La Jolla, CA, USA). Two independent groups were compared via using Student’s *t*-test. The one-way analysis of variance was utilized to assess the difference for more than two groups. The correlation among miR-182-5p, hsa_circ_0007121, and PGF in PE placental tissues was analyzed by Pearson’s correlation coefficient. Each experiment was carried out with at least three replications. *P* < 0.05 indicated the statistical significance.

## 3 Results

### 3.1 Hsa_circ_0007121 is downregulated in PE placental tissues

**Figure 1:**
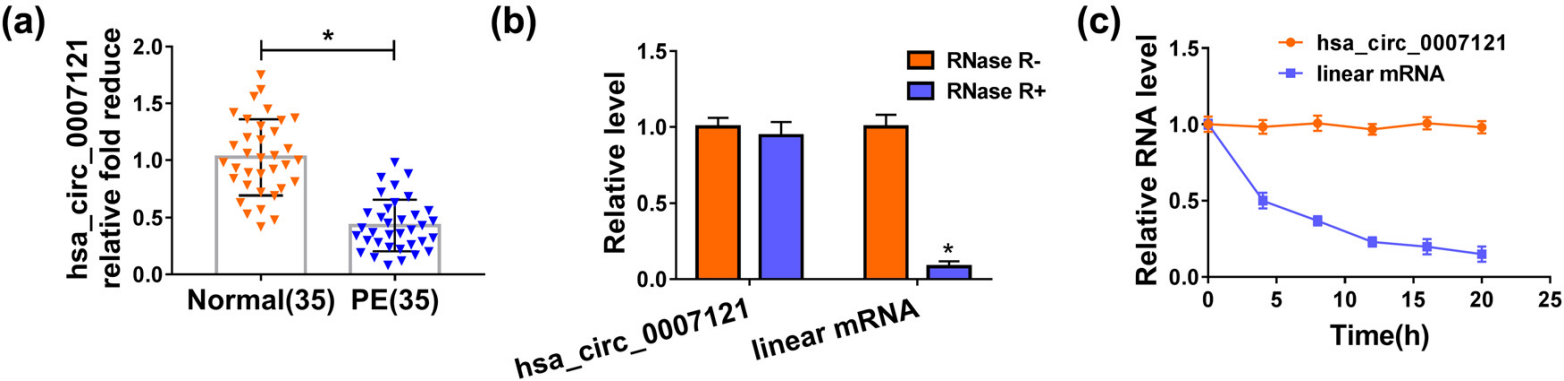
Hsa_circ_0007121 was downregulated in PE placental tissues. (a) The level of hsa_circ_0007121 in PE placental tissues (*n* = 35) and normal placental tissues (*n* = 35) was measured by qRT-PCR. (b) Hsa_circ_0007121 and the linear mRNA levels in HTR-8/SVneo cells treated with or without RNase R were detected by qRT-PCR. (c) hsa_circ_0007121 and the linear mRNA levels in HTR-8/SVneo cells treated with actinomycin D at the pointed time were checked by qRT-PCR. *P < 0.05.

First, we measured hsa_circ_0007121 level in PE placental tissues and compared them with those in normal placentas. The results showed that relative to the normal placental tissues, hsa_circ_0007121 was significantly downregulated in PE placental tissues (Figure 1a). Then, the levels of hsa_circ_0007121 and the linear mRNA were checked, and the data indicated that hsa_circ_0007121 level was not clearly changed under treatment with RNase R, while the level of linear mRNA was apparently declined under RNase R treatment (Figure 1b). Besides, the transcript half-life of hsa_circ_0007121 (nearly 20 h) was longer than the half-life of linear mRNA (less than 5 h) after the treatment with actinomycin D (Figure 1c). These data suggested that hsa_circ_0007121 was downregulated with high stability in HTR-8/SVneo cells than linear mRNA.

### 3.2 Hsa_circ_0007121 regulated HTR-8/SVneo cell proliferation, migration, invasion, and EMT

**Figure 2:**
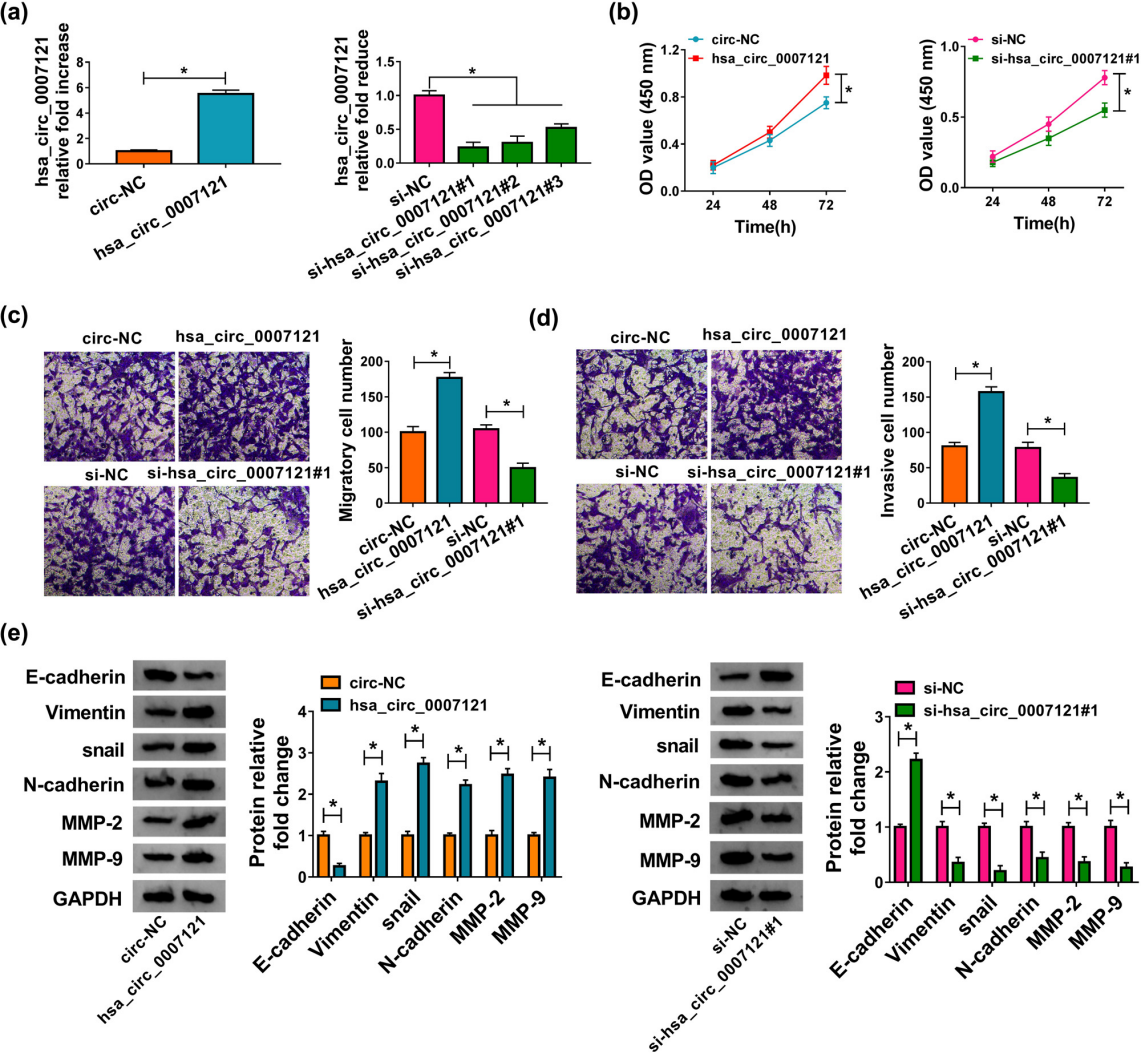
Hsa_circ_0007121 was involved in the regulation of PE. (a) The overexpression efficiency of hsa_circ_0007121 and the knockdown efficiency of si-hsa_circ_0007121 (si-hsa_circ_0007121#1, si-hsa_circ_0007121#2, and si-hsa_circ_0007121#3) were evaluated by qRT-PCR. (b) The proliferation of HTR-8/SVneo cells transfected with circ-NC, hsa_circ_0007121, si-NC, or si-hsa_circ_0007121#1 was checked by the CCK8 assay. (c and d) Cell migration and invasion were checked by the transwell assay. (e) The protein levels of EMT-related proteins (E-cadherin, Vimentin, snail, N-cadherin, MMP-2, and MMP-9) in transfected HTR-8/SVneo cells were measured by western blot assay. *P < 0.05.

The effect of hsa_circ_0007121 on PE was further investigated, and we detected its level in HTR-8/SVneo cells after transfection with circ-NC, hsa_circ_0007121, si-NC, or si-hsa_circ_0007121 (si-hsa_circ_0007121#1, si-hsa_circ_0007121#2, and si-hsa_circ_0007121#3). The result showed that hsa_circ_0007121 was conspicuously upregulated in hsa_circ_0007121 group relative to circ-NC group, and it was significantly downregulated in the si-hsa_circ_0007121 group compared with the si-NC group (Figure 2a). Overexpression of hsa_circ_0007121 promoted cell proliferation, while an opposite result was obtained when hsa_circ_0007121 was knocked down (Figure 2b). Meanwhile, the transwell assay indicated that cell migration and invasion were boosted by upregulated hsa_circ_0007121, while repressed by downregulation of hsa_circ_0007121 (Figure 2c and d). Moreover, EMT-related protein levels were checked, and the results revealed that hsa_circ_0007121 overexpression reduced the level of E-cadherin and elevated the levels of Vimentin, snail, N-cadherin, MMP2, and MMP9, while hsa_circ_0007121 silencing exhibited opposite results (Figure 2e). On the whole, these results illustrated that hsa_circ_0007121 was involved in the modulation of PE progression.

### 3.3 Hsa_circ_0007121 directly targeted miR-182-5p to regulate its expression

**Figure 3:**
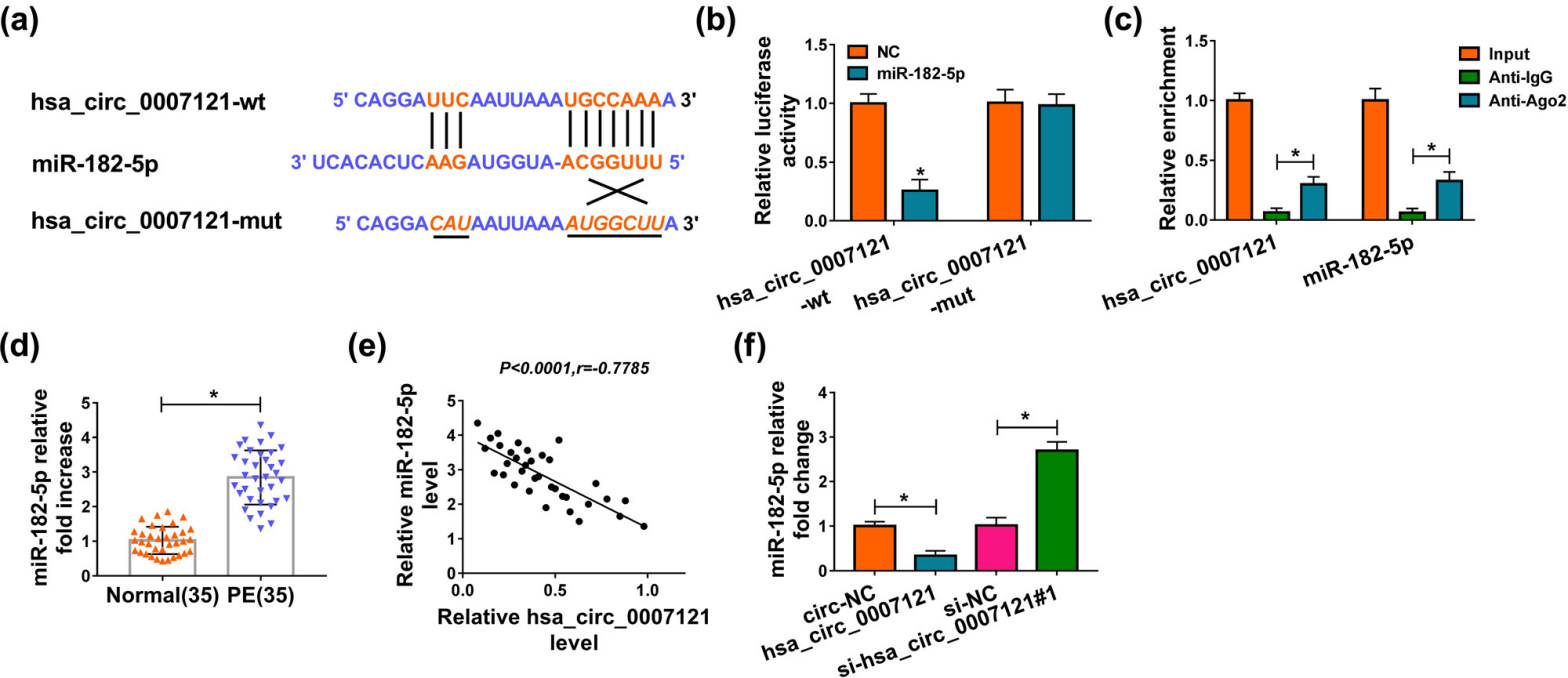
Hsa_circ_0007121 directly interacted with miR-182-5p. (a) The potential target sites between miR-182-5p and hsa_circ_0007121 were predicated by CircRNA interactome. (b and c) The dual-luciferase reporter assay and RIP assay were performed to investigate the interaction between miR-182-5p and hsa_circ_0007121. (d) The level of miR-182-5p in PE placental tissues (*n* = 35) and normal placental tissues (*n* = 35) was detected by qRT-PCR. (e) The correlation between miR-182-5p and hsa_circ_0007121 in PE placental tissues was analyzed by Pearson’s correlation coefficient. (f) The expression level of miR-182-5p was measured by qRT-PCR in HTR-8/SVneo cells transfected with circ-NC, hsa_circ_0007121, si-NC, or si-hsa_circ_0007121#1. *P < 0.05.

To explore how hsa_circ_0007121 participates in the modulation of PE progression, CircRNA interactome was used to explore its potential target, and we found that hsa_circ_0007121 contained the complementary sequences of miR-182-5p, which suggested that miR-182-5p might be bound to hsa_circ_0007121 (Figure 3a). Then, the luciferase activity of hsa_circ_0007121-wt in HTR-8/SVneo cells was notably diminished by miR-182-5p, while there was no change in the hsa_circ_0007121-mut group (Figure 3b). Besides, RIP assay exhibited that both hsa_circ_0007121 and miR-182-5p were enriched when incubation with Anti-Ago2 in comparison to Anti-IgG (Figure 3c). Next, miR-182-5p level was checked, and we found that it was strikingly higher in PE placental tissues than that in normal placental tissues (Figure 3d). Moreover, miR-182-5p was negatively associated with hsa_circ_0007121 in PE placental tissues (Figure 3e). In addition, hsa_circ_0007121 overexpression significantly decreased the level of miR-182-5p in HTR-8/SVneo cells, whereas hsa_circ_0007121 knockdown evidently increased the levels miR-182-5p (Figure 3f). Collectively, these results illustrated that hsa_circ_0007121 negatively regulated miR-182-5p via directly targeting.

### 3.4 Hsa_circ_0007121 regulated HTR-8/SVneo cell proliferation, migration, invasion, and EMT through miR-182-5p

**Figure 4:**
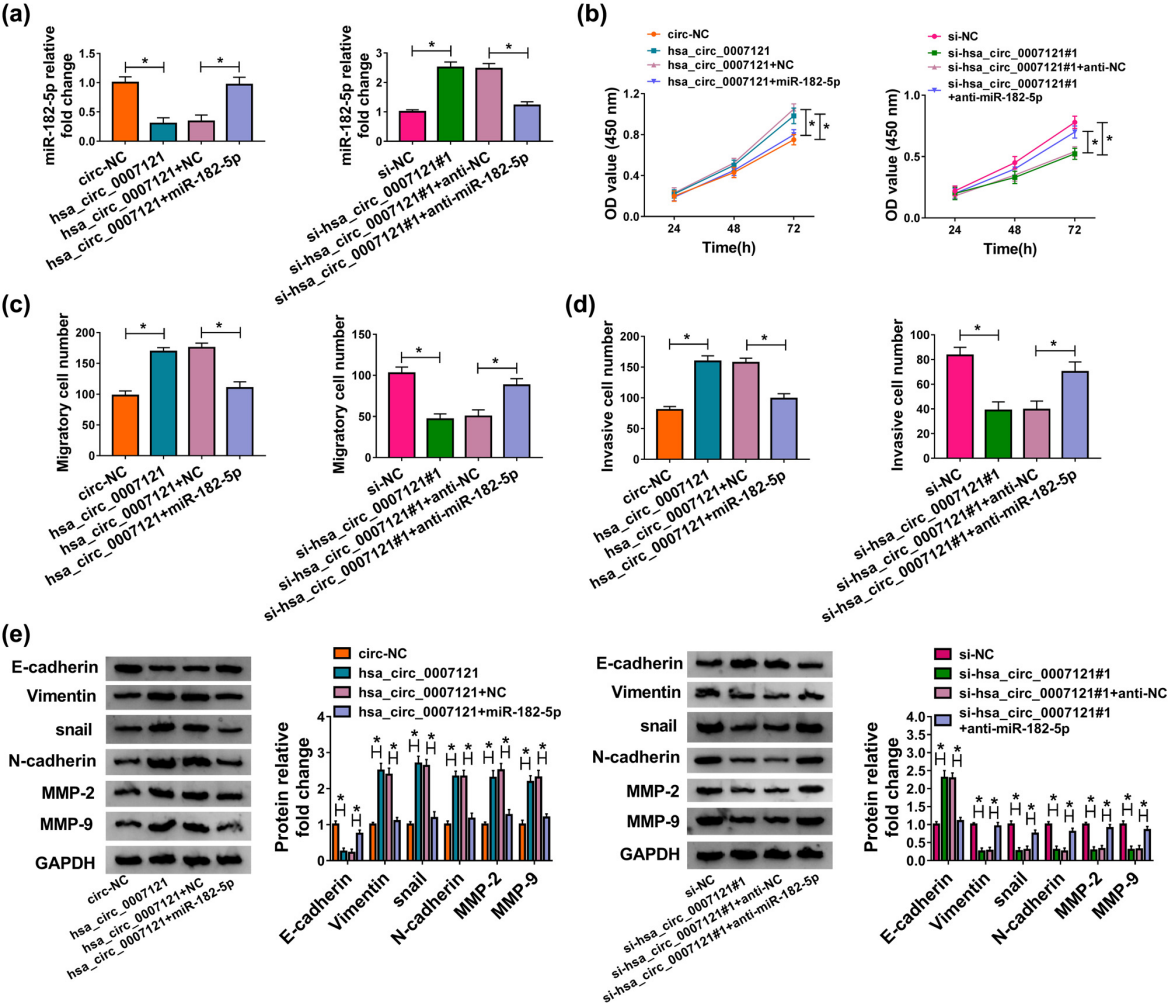
Hsa_circ_0007121 regulated PE progression by interacting with miR-182-5p. (a) The level of miR-182-5p in HTR-8/SVneo cells transfected with hsa_circ_0007121, hsa_circ_0007121 + miR-182-5p, si-hsa_circ_0007121#1, or si-hsa_circ_0007121#1 + anti-miR-182-5p, as well as matched controls was checked by qRT-PCR. (b) The proliferation of transfected HTR-8/SVneo cells was checked by the CCK8 assay. (c and d) The abilities of migration and invasion of transfected HTR-8/SVneo cells were estimated by the transwell assay. (e) The protein levels of EMT-related proteins in samples were detected by Western blot. *P < 0.05.

To investigate the functional mechanism between hsa_circ_0007121 and miR-182-5p, HTR-8/SVneo cells were transfected with hsa_circ_0007121, hsa_circ_0007121 + miR-182-5p, si-hsa_circ_0007121#1, or si-hsa_circ_0007121#1 + anti-miR-182-5p, as well as matched controls. QRT-PCR result shows that the expression of miR-182-5p was inhibited in the cell transfected hsa_circ_0007121, while this inhibition effect was reversed when miR-182-5p was upregulated; meanwhile anti-miR-182-5p reversed the promotion effect on miR-182-5p expression induced by circ_0007121 knockdown (Figure 4a). Subsequently, CCK-8 results exhibited that upregulation of miR-182-5p reversed the promotion effect on cell proliferation induced by hsa_circ_0007121 overexpression, and miR-182-5p knockdown overturned hsa_circ_0007121 silencing-mediated inhibitory effect on cell proliferation (Figure 4b). Besides, the transwell assay indicated that miR-182-5p mimic rescued hsa_circ_0007121 overexpression induced migration and invasion, and its inhibitor inverted the inhibited migration and invasion caused by hsa_circ_0007121 knockdown (Figure 4c and d). Moreover, the levels of EMT-related proteins in the hsa_circ_0007121 group or the si-hsa_circ_0007121 group were reversely changed after miR-182-5p was overexpressed or knockdown, respectively (Figure 4e). In general, these findings disclosed that hsa_circ_0007121 regulated PE development by targeting miR-182-5p.

### 3.5 Hsa_circ_0007121 regulated PGF expression via targeting miR-182-5p

**Figure 5:**
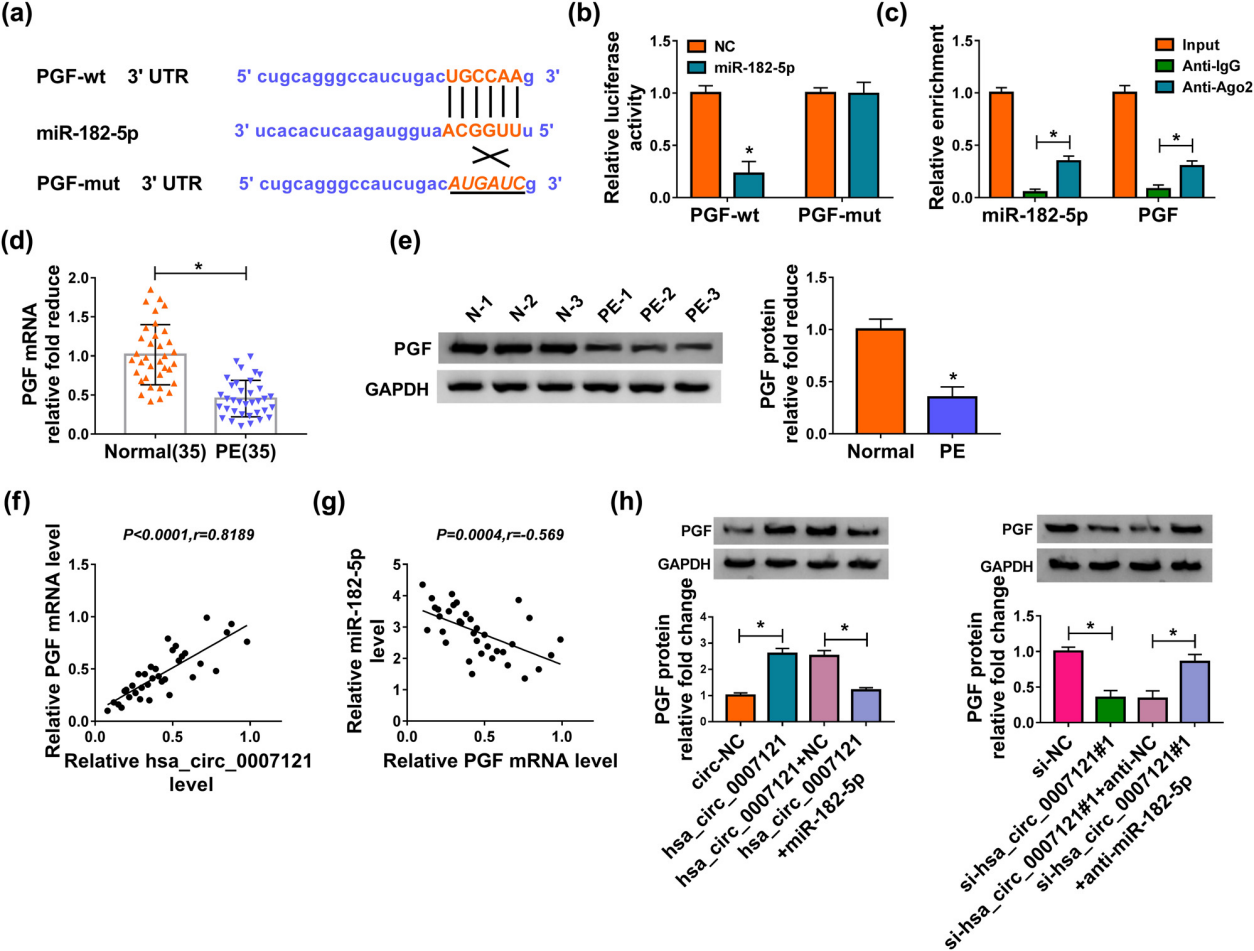
MiR-182-5p bound to the 3’UTR of PGF and negatively regulated PGF expression. (a) The putative binding sites between miR-182-5p and PGF were predicated by ENCORI. (b and c) The interaction between miR-182-5p and PGF was explored by the dual-luciferase reporter assay and RIP assay. (d and e) The mRNA and the protein levels of PGF in PE placental tissues (*n* = 35) and normal placental tissues (*n* = 35) were checked by qRT-PCR and western blot, respectively. (f and g) The correlation between PGF and hsa_circ_0007121 or miR-182-5p in PE placental tissues was analyzed using Pearson’s correlation coefficient. (h) The protein level of PGF in HTR-8/SVneo cells transfected with hsa_circ_0007121, hsa_circ_0007121 + miR-182-5p, si-hsa_circ_0007121#1, or si-hsa_circ_0007121#1 + anti-miR-182-5p, as well as corresponding controls was checked by qRT-PCR. *P < 0.05.

ENCORI was used to find the possible targets of miR-182-5p. It was displayed that the existence of binding sites between miR-182-5p and PGF 3’UTR (Figure 5a), and the dual-luciferase reporter assay and RIP assay further verified this interaction (Figure 5b and c). We then discovered that the PGF level was clearly decreased in PE placental tissues (Figure 5d and e). Moreover, PGF mRNA level was positively associated with hsa_circ_0007121 (Figure 5f) and had an opposite correlation with miR-182-5p in PE placental tissues (Figure 5g). Further analysis demonstrated that the elevated protein level of PGF in the hsa_circ_0007121 group was reversed when miR-182-5p overexpressed, and the decreased protein level of PGF in the si-hsa_circ_0007121 group was also inverted by miR-182-5p inhibitor (Figure 5h). Our findings indicated that PGF was a target of miR-182-5p- and hsa_circ_0007121-modulated PGF expression via miR-182-5p.

### 3.6 MiR-182-5p-/PGF axis-modulated HTR-8/SVneo cell proliferation, migration, invasion, and EMT

**Figure 6:**
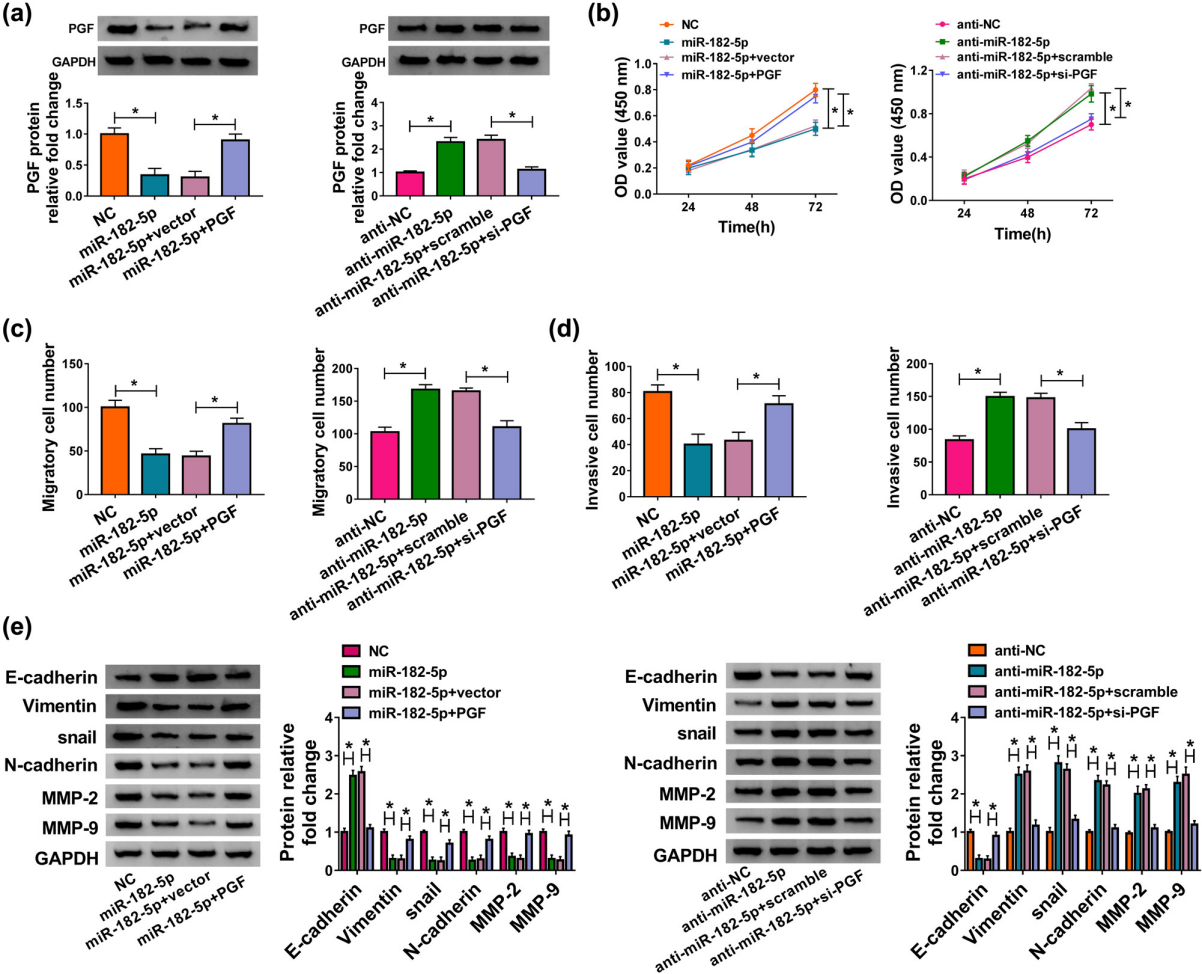
MiR-182-5p regulated PE progression via targeting PGF. (a) The protein level of PGF in HTR-8/SVneo cells transfected with miR-182-5p, miR-182-5p + PGF, anti-miR-182-5p, and anti-miR-182-5p + si-PGF was measured by western blot. (b) The CCK8 assay was conducted to check the proliferation of transfected HTR-8/SVneo cells. (c and d) The transwell assay was executed to evaluate the abilities of cell migration and invasion. (e) The protein levels of EMT-related proteins were determined by western blot. *P < 0.05.

To dissect the mechanism of miR-182-5p and PGF in PE progression, we first measured the protein level of PGF in transfected HTR-8/SVneo cells. The declined protein level of PGF was observed in the miR-182-5p group, and this trend was reversed by PGF overexpression, and the increased protein level of PGF due to anti-miR-182-5p was reversed following the transfection with si-PGF (Figure 6a). CCK8 assay showed that PGF overexpression inverted miR-182-5p upregulation-mediated suppressive cell proliferation, while the promoted cell proliferation due to miR-182-5p downregulation was recovered by PGF knockdown (Figure 6b). Meanwhile, miR-182-5p overexpression weakened migration and invasion was receded by PGF overexpression, and PGF silencing revoked miR-182-5p depletion-mediated boosted effects on cell migration and invasion (Figure 6c and d). Analogously, upregulation of PGF rescued the effect of miR-182-5p on EMT, whereas downregulation of PGF rescued miR-182-5p depletion-mediated promoted effect on EMT (Figure 6e). These data demonstrated that miR-182-5p targeted PGE to regulate PE development.

## 4 Discussion

PE is a growing threat to the pregnant woman, and nearly, 76,000 pregnant women died from PE and related hypertensive disorders every year [35]. Therefore, it is a crying need for exploring the underlying mechanism and discovering new therapeutic strategies for PE. The previous research showed that circRNAs were closely related to the regulation of PE. Deng et al. confirmed that hsa_circ_0011460 might serve as a biomarker for the treatment of severe PE [13]. Zhou et al. reported that knockdown of circPAPPA facilitated the onset and development of PE via inhibiting trophoblast cells [14]. hsa_circ_0007021, which was found to be decreased in PE plasma before the disease phenotype presents, might be a novel biomarker of preeclampsia [16]. In our study, hsa_circ_0007121 level was reduced in PE placental tissues compared with the normal placental tissues, which was in line with the previous report [16]. Here, we first proposed the regulatory network of hsa_circ_0007121/miR-182-5p/PGF and revealed the effect and underlying mechanisms of hsa_circ_0007121 in PE.

Growing evidence have elucidated that circRNAs act as a competing endogenous RNA (ceRNA) and could also sponging miRNAs to regulate the expression of the downstream genes. Wu et al. reported that circTADA2A promoted cell proliferation and metastasis in osteosarcoma by binding to miR-203a-3p [36]. Wang et al. reported that circRNA MFACR modulated cardiomyocyte death by sponging miR-652-3p [37]. In this research, miR-182-5p was confirmed to be bound and negatively regulated by hsa_circ_0007121. Besides, miR-182-5p overexpression or knockdown reversed hsa_circ_0007121 upregulation- or silencing-mediated effect on HTR-8/SVneo cell proliferation, migration, invasion, and EMT, indicating that hsa_circ_0007121 plays roles in PE development by regulating miR-182-5p.

To further understand the mechanism of miR-182-5p in regulating PE, we predicated and verified its target gene, PGF, which was tightly associated with PE [25,31,32]. In this study, we found a decreased expression of PEG in PE placental tissues, which was in accordance with a recent report [33]. In addition, PGF was positively correlated with hsa_circ_0007121 and negatively associated with miR-182-5p in PE placental tissues. Moreover, hsa_circ_0007121 altered PGF expression via sponging miR-182-5p. Also, PGF overexpression or downregulation rescued miR-182-5p mimic- or inhibitor-mediated impact on proliferation, migration, invasion, and EMT in HTR-8/SVneo cells. Therefore, these results suggested that hsa_circ_0007121 could regulate the expression of PGF by sponging miR-182-5p, eventually influencing the progression of PE. Although our research provides the theoretical support for the application of hsa_circ_0007121 in PE therapy, other function of has_circ_007121 in PE still needs further exploration, and animal model of PE is required for further study to better elucidate the mechanism of hsa_circ_0007121 in PE.

In conclusion, our studies suggested that hsa_circ_0007121 was notably downregulated in PE placental tissues and HTR-8/SVneo cells, and hsa_circ_0007121 mediated the EMT of trophoblast cells proliferation, migration, invasion, and EMT via miR-182-5p/PGF axis. This novel mechanism might provide a new light for the therapy of PE.
